# Supplementary material for: Kynurenine aminotransferase II inhibition promotes sleep and rescues impairments induced by neurodevelopmental insult
Source: Transl Psychiatry. 2023 Mar 31;13:106. doi: 10.1038/s41398-023-02399-1 (PMC10066394; doi:10.1038/s41398-023-02399-1)
Supplement: Supplementary file 1 — Milosavljevic et al Supplementary Information [file 41398_2023_2399_MOESM1_ESM.docx]

TITLE: **Kynurenine aminotransferase II inhibition promotes sleep and rescues impairments induced by neurodevelopmental insult**

**Authors**: Snezana Milosavljevic, PhD^1^, Andrew K. Smith, BS^2^, Courtney J. Wright, BS^1^, Homayoun Valafar, PhD^2^, Ana Pocivavsek, PhD^1^

**Affiliations**:

^1^Department of Pharmacology, Physiology and Neuroscience, University of South Carolina School of Medicine, Columbia, South Carolina

^2^Department of Computer Science and Engineering, University of South Carolina, Columbia, South Carolina

**Corresponding Author**:

Ana Pocivavsek, Ph.D.

University of South Carolina School of Medicine

Department of Pharmacology, Physiology and Neuroscience

Building 1, D26

6311 Garners Ferry Rd

Columbia, SC 29209, USA

Phone: (803) 216-3509

Fax: (803) 216-3554

ana.pocivavsek@uscmed.sc.edu


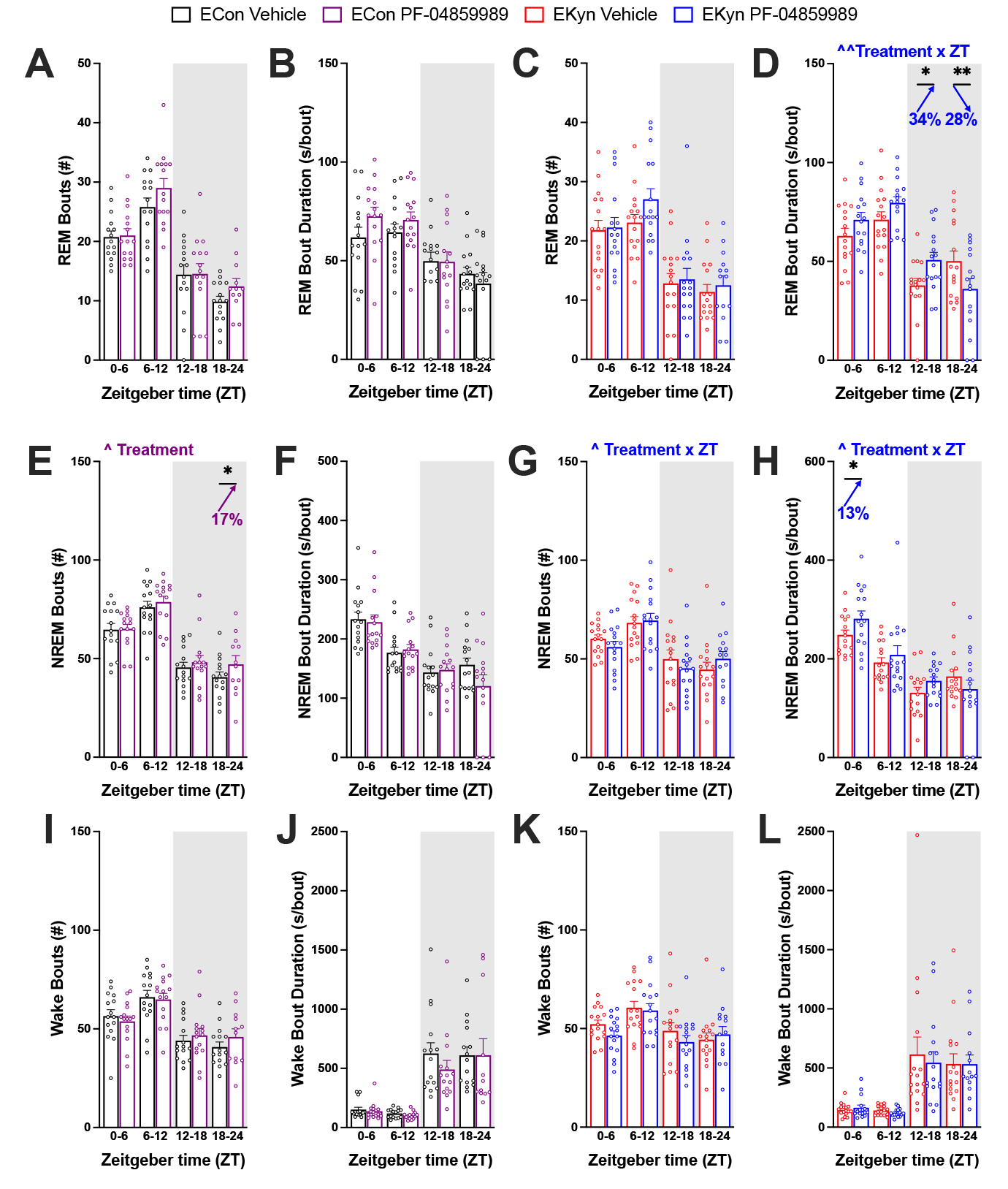


**Supplementary Figure 1. Sleep architecture following inhibition of KYNA synthesis at the beginning of light phase.** Adult ECon and EKyn offspring were treated with vehicle or PF-04859989 (30 mg/kg) at Zeitgeber time (ZT) 0. **(A)** 6-hr bins of number of REM bouts in ECon, sexes combined. **(B)** 6-hr bins of average REM bout duration in ECon, sexes combined. **(C)** 6-hr bins of number of REM bouts in EKyn, sexes combined. **(D)** 6-hr bins of average REM bout duration in EKyn, sexes combined (Treatment x ZT interaction F_(3, 45)_= 6.024, P<0.01). **(E)** 6-hr bins of number of NREM bouts in ECon, sexes combined. **(F)** 6-hr bins of average NREM bout duration in ECon, sexes combined. **(G)** 6-hr bins of number of NREM bouts in EKyn, sexes combined (Treatment x ZT interaction F_(3, 43)_= 3.471, P<0.05). **(H)** 6-hr bins of average NREM bout duration in EKyn, sexes combined (Treatment x ZT interaction F_(3, 45)_= 3.973, P<0.05). **(I)** 6-hr bins of number of wake bouts in ECon, sexes combined. **(J)** 6-hr bins of average wake bout duration in ECon, sexes combined. **(K)** 6-hr bins of number of wake bouts in EKyn, sexes combined. **(L)** 6-hr bins of average wake bout duration in EKyn, sexes combined. Data are mean ± SEM. Percent change from vehicle treatment calculations are shown by arrows. Two-way RM ANOVA: ^P<0.05, ^^P<0.01 with Fisher’s LSD post hoc test: *P<0.05, **P<0.01. N = 12-16 per group.

**
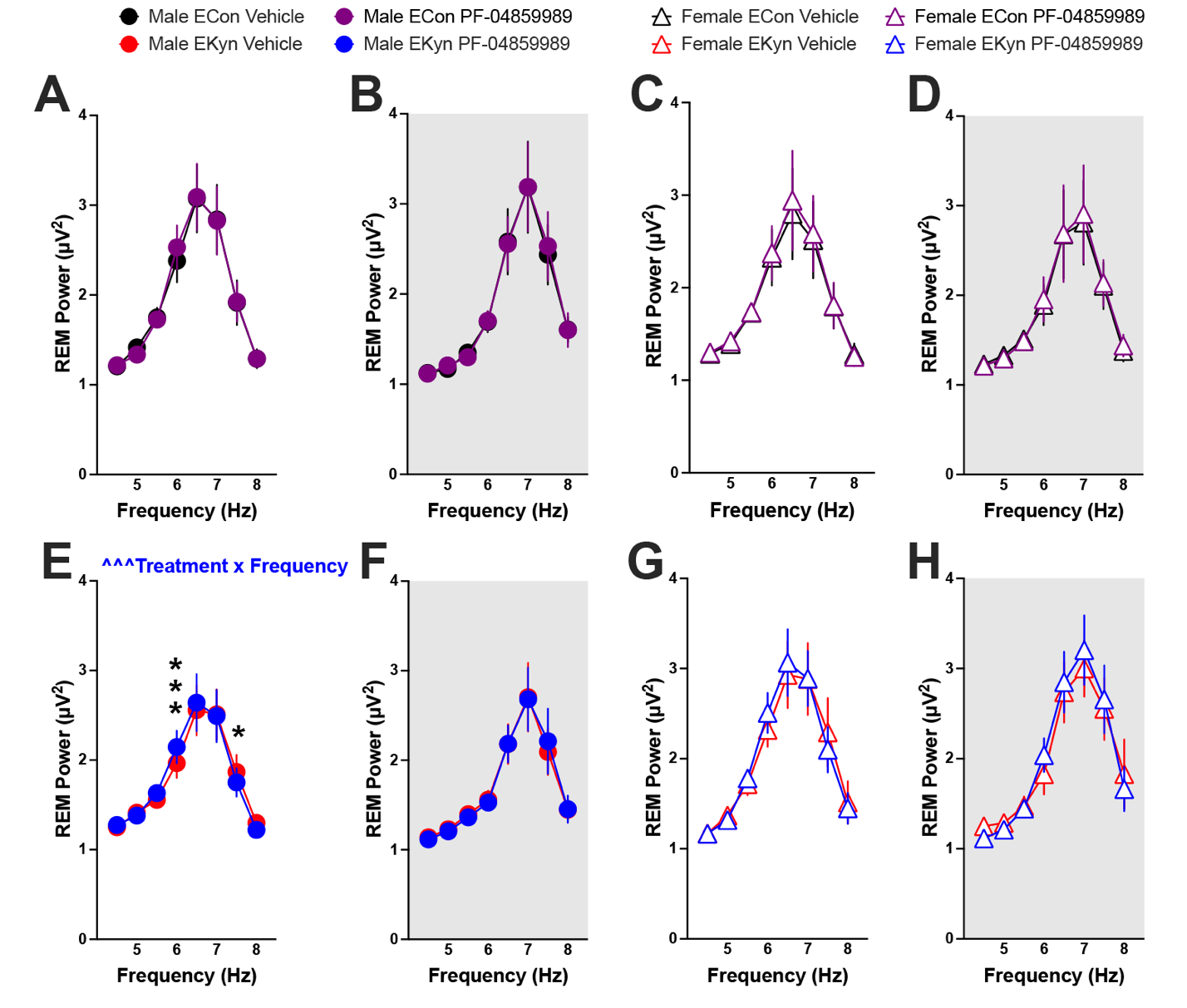
Supplementary Figure 2. REM theta spectral power after reduction in KYNA levels at the beginning of light phase.** Adult ECon and EKyn offspring were treated with vehicle or PF-04859989 (30 mg/kg) at Zeitgeber time (ZT) 0. **(A)** Male ECon during light phase. **(B)** Male ECon during dark phase. **(C)** Female ECon during light phase. **(D)** Female ECon during dark phase. **(E)** Male EKyn during light phase (Treatment x Frequency interaction F_(7, 42)_= 4.738, P<0.001). **(F)** Male EKyn during dark phase. **(G)** Female EKyn during light phase. **(H)** Female EKyn during dark phase. Data are mean ± SEM. Two-way RM ANOVA: ^^^P<0.001 with Fisher’s LSD post hoc test: *P<0.05, ***P<0.001. N = 7-8 per group.


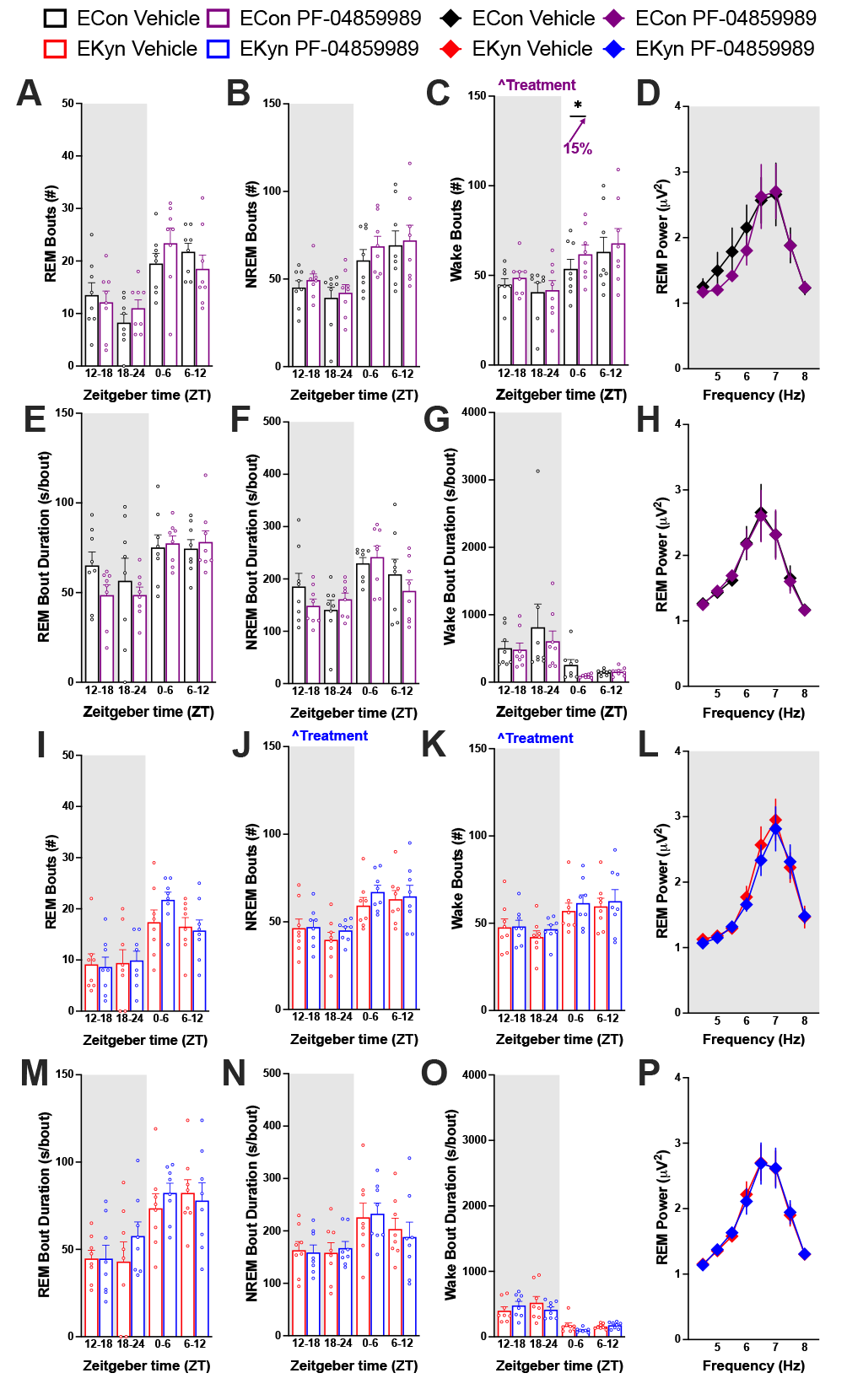


**Supplementary Figure 3. Sleep architecture following KAT II inhibition at the beginning of dark phase.** Adult ECon and EKyn offspring were treated with vehicle or PF-04859989 (30 mg/kg) at Zeitgeber time (ZT) 12. **(A)** 6-hr bins of number of REM bouts in ECon, sexes combined. **(B)** 6-hr bins of number of NREM bouts in ECon, sexes combined. **(C)** 6-hr bins of number of wake bouts in ECon, sexes combined (Treatment effect F_(1, 7)_= 6.334, P<0.05). **(D)** REM theta spectral power during dark phase in ECon, sexes combined. **(E)** 6-hr bins of average REM bout duration in ECon, sexes combined. **(F)** 6-hr bins of average NREM bout duration in ECon, sexes combined. **(G)** 6-hr bins of average wake bout duration in ECon, sexes combined. **(H)** REM theta spectral power during light phase in ECon, sexes combined. **(I)** 6-hr bins of number of REM bouts in EKyn, sexes combined. **(J)** 6-hr bins of number of NREM bouts in EKyn, sexes combined (Treatment effect F_(1, 7)_= 6.975, P<0.05). **(K)** 6-hr bins of number of wake bouts in EKyn, sexes combined (Treatment effect F_(1, 7)_= 5.826, P<0.05). **(L)** REM theta spectral power during dark phase in EKyn, sexes combined. **(M)** 6-hr bins of average REM bout duration in EKyn, sexes combined. **(N)** 6-hr bins of average NREM bout duration in EKyn, sexes combined. **(O)** 6-hr bins of average wake bout duration in EKyn, sexes combined. **(P)** REM theta spectral power during light phase in EKyn, sexes combined. Data are mean ± SEM. Percent change from vehicle treatment calculations are shown by arrows. Two-way RM ANOVA: ^P<0.05 with Fisher’s LSD post hoc test: *P<0.05. N = 8 per group.


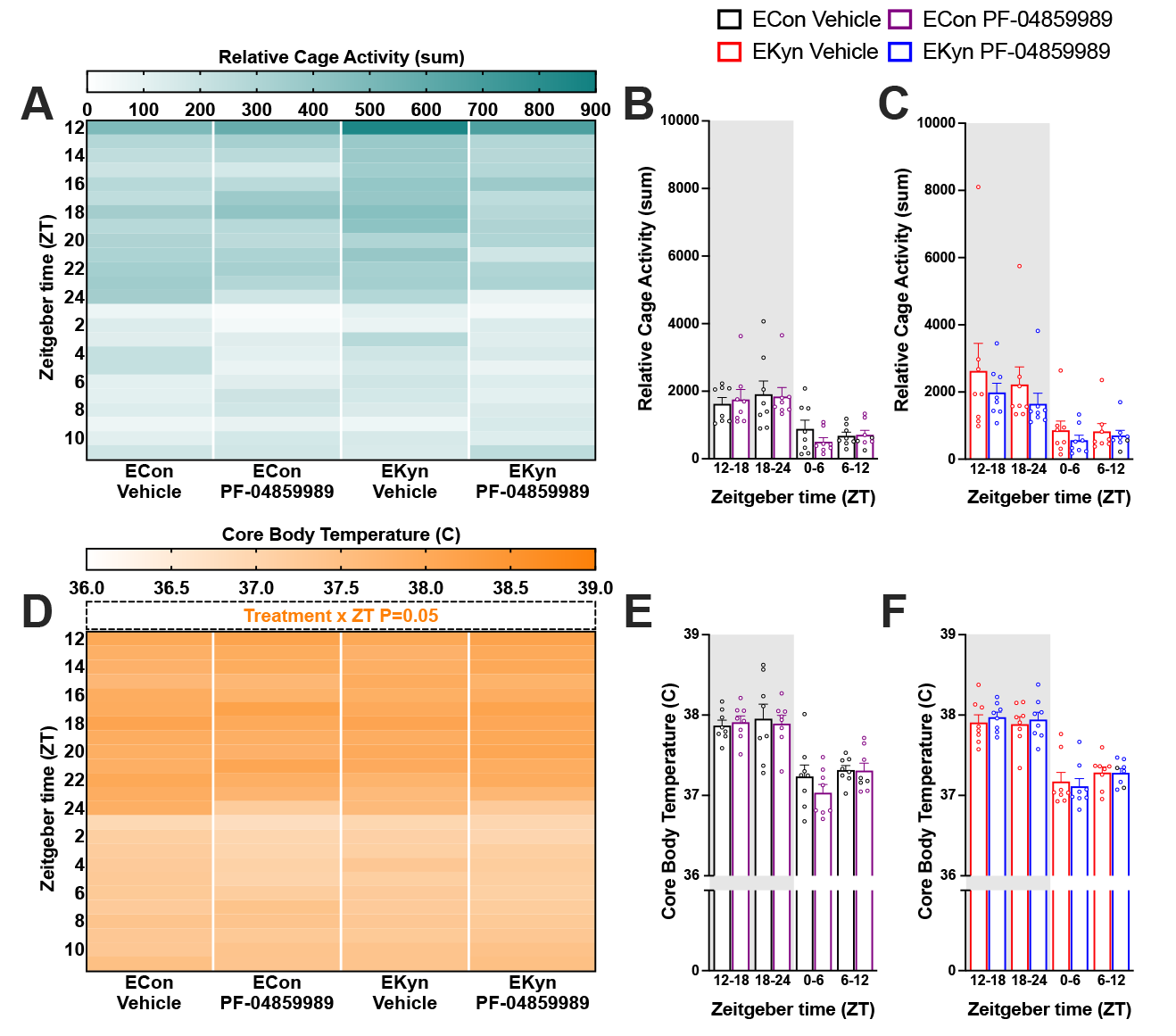


**Supplementary Figure 4. Activity and temperature after administration of KAT II inhibitor at the beginning of dark phase.** Adult ECon and EKyn offspring were treated with vehicle or PF-04859989 (30 mg/kg) at Zeitgeber time (ZT) 12. **(A)** 1-hr bins of relative cage activity, sexes combined. **(B)** 6-hr bins of relative cage activity in ECon, sexes combined. **(C)** Relative cage activity in EKyn, sexes combined. **(D)** 1-hr bins of core body temperature, sexes combined. **(E)** 6-hr bins of core body temperature in ECon, sexes combined. **(F)** Core body temperature in EKyn, sexes combined. Data are mean ± SEM. N = 11-16 per group.

**
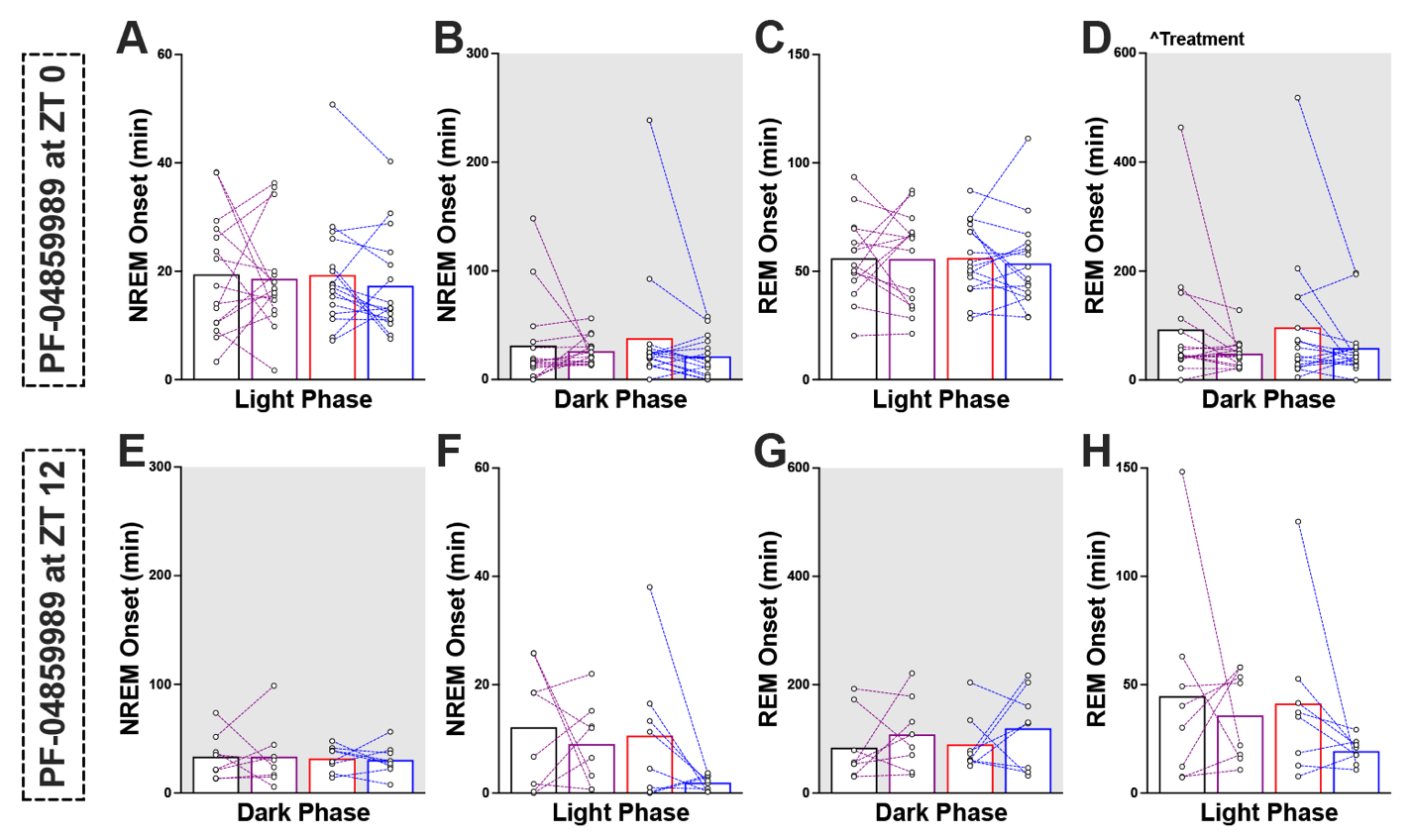
Supplementary Figure 5. NREM and REM onset following PF-04859989 administration.** Adult ECon and EKyn offspring were treated with vehicle or PF-04859989 (30 mg/kg) at Zeitgeber time (ZT) 0 or ZT 12. **(A)** NREM onset in light phase, sexes combined. **(B)** NREM onset in dark phase, sexes combined. **(C)** REM onset in light phase, sexes combined. **(D)** REM onset in dark phase, sexes combined (Treatment effect F_(1, 29)_= 4.924, P<0.05). **(E)** NREM onset in dark phase, sexes combined. **(F)** NREM onset in light phase, sexes combined. **(G)** REM onset in dark phase, sexes combined. **(H)** REM onset in light phase, sexes combined. Data are mean ± SEM. Two-way RM ANOVA: ^P<0.05. N = 8-16 per group.

**
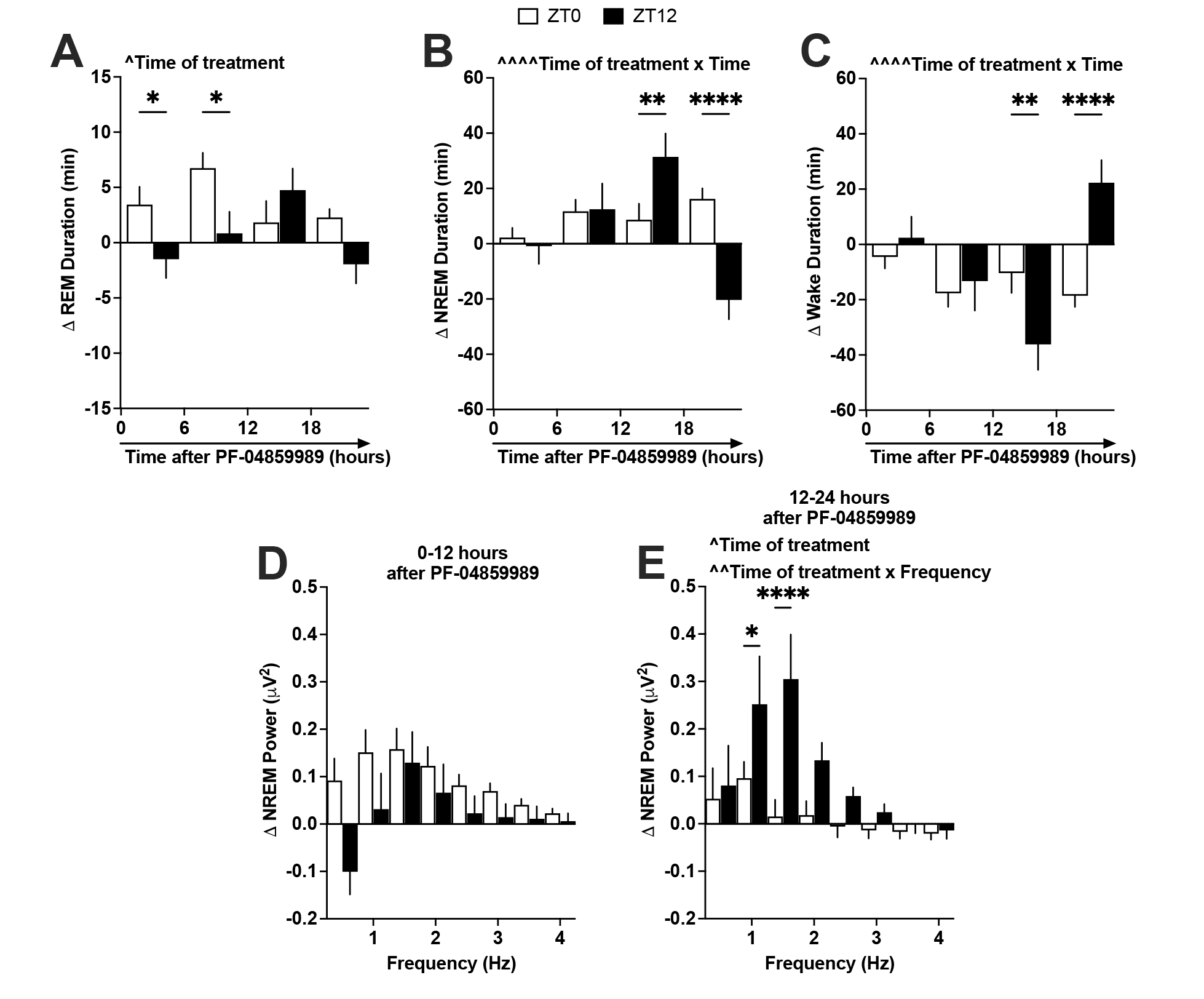
Supplementary Figure 6. Comparison of changes in sleep architecture between PF-04859989 administration at ZT 0 or ZT 12.** Adult ECon and EKyn offspring were treated with vehicle or PF-04859989 (30 mg/kg) at Zeitgeber time (ZT) 0 or ZT 12. **(A)** 6-hr bins of absolute change in REM duration, sexes and prenatal conditions combined (Time of treatment effect F_(1, 175)_= 5.935, P<0.05). **(B)** 6-hr bins of absolute change in NREM duration, sexes and prenatal conditions combined (Time of treatment x Time interaction F_(3, 130)_= 8.322, P<0.0001). **(C)** 6-hr bins of absolute change in wake duration, sexes and prenatal conditions combined (Time of treatment x Time interaction F_(3, 130)_= 7.712, P<0.0001). **(D)** Absolute change in NREM delta spectral power 0-12 hours after PF-04859989, sexes and prenatal conditions combined. **(E)** Absolute change in NREM delta spectral power 12-24 hours after PF-04859989, sexes and prenatal conditions combined (Time of treatment effect F_(1, 45)_= 6.064, P<0.05, Time of treatment x Frequency interaction F_(7, 315)_= 3.078, P<0.01). Data are mean ± SEM. Two-way RM ANOVA: ^P<0.05, ^^^^P<0.0001 with Fisher’s LSD post hoc test: *P<0.05, **P<0.01, ****P<0.0001. N = 16-31 per group.
